# Intelligence, Correspondence, &c.

**Published:** 1832-04-01

**Authors:** 


### INTELLIGENCE, CORRESPONDENCE, &c.

The letter of J. M. C. is received, and he is requested to use his own discretion as to the *extent* of the paper on which he is employed.

A " constant reader's" case of sudden death, where the internal surface of the ventricles and also of the aorta was of a " brilliant vermillion colour," has been received and perused. We cannot deny that the colour in the above case was from injection of the vessels, nor assert that it resulted from imbibition. The heart is reported to have been " small and flabby," and as sudden death is not unusual in such conditions of the organ, we have hesitation in attributing the fatal event to the state of the lining membrane of the heart and aorta.

The fasciculus required by Mr. Heys near Wisbeach, is forwarded to Messrs. Rivington, as directed by Mr. H.

Mr. Green's gazeous mercurial inhaler, together with his apparatus for the hot air-bath, must be seen at his own house, as we cannot give a description of them.

